# Practical Notes on Diagnosis and Treatment

**Published:** 1906-12-15

**Authors:** 


### Practical Notes on Diagnosis and Treatment.

**Antipyrin in Epistaxis.**

Dr. E. G. West finds antipyrin, used as a local application, of value in cases of severe epistaxis. He saturates a pledget of cotton-wool with a solution of the drug, and introduces this into the nostril, or sometimes he applies the powder itself on the wool.

**Treatment of Psoriasis.**

Arsenic should not be given when there is much congestion of the skin. In such a case large doses of potassium iodide (20 increased to 60 grains in a bitter infusion) thrice daily are indicated. Whatever local remedies are employed they should be persevered with until all traces of the eruption have disappeared. Even then a modified arsenical treatment should be continued.—*Dr. J. J. Pringle.*

**Paraplegia in Syphilis.**

The great majority of patients who suffer from this condition recover (under vigorous treatment by specifics) up to a certain point and regain the power of walking. The improvement is, however, never progressive after a certain comparatively short period has elapsed. A very cheering point in reference to the prognosis is that no relapses occur.—*Mr. Jonathan Hutchinson.*

**Atony of the Bladder.**

Tincture of nux vomica is the routine drug for this condition, and it occasionally proves useful. When it fails, liquid extract of ergot should be ordered—say 10 minims thrice daily. This may restore the power of the bladder even in patients who have been for weeks dependent on the catheter. The same drug is valuable in the hæmaturia associated with enlargement of the prostate.—*Mr. Reginald Harrison.*

**Hæmoptysis in Aneurism.**

Slight hæmoptysis, continuing for several weeks, is not inconsistent with a diagnosis of thoracic aneurism. In fact, such a condition, if not otherwise explained, rather suggests a hidden aneurism of the aorta. It is a mistake to think that an aneurism usually bursts suddenly into the trachea or into a bronchus. As a matter of fact, communication with the air passages is usually established by ulceration, and this permits repeated leakages of blood.

